# Development and Structure of the Human Teeth

**Published:** 1859-10

**Authors:** 


*Development and Structure of the Human Teeth.*—We complete, in the present number of the Journal, the publication of the translation of this highly interesting monograph. M. Magitot, the author, deserves, and will receive the thanks, not only of Odontologists, but also of every scientific member of the dental profession, for this very able contribution to dental science. Although we cannot agree with him as to the precise manner of the formation of the dental tissues, we do not value the treatise any the less on that account, and we have no hesitation in saying that it is one of the ablest and most ingenious works that has, for a long time, issued from the press on the subject. We earnestly recommend a careful and thorough perusal of it to each of our readers, feeling assured that the study will be productive both of pleasure and profit. If any class of men should be thoroughly acquainted with the teeth, surely it is dentists.

But, comparatively speaking, and I am sorry to say it, very few have made the structure of these organs a subject of careful study.

The illustrative engravings of the original work should appear at the end of the treatise in the present number of the Journal. They were placed in the hands of a lithographer, in July last. He promised that they should be completed by the first of September, but on calling on him late in August to ascertain if he had executed the work, we were informed that after undertaking to do it, he found he could not and had entrusted it to another individual, who stated that it would be impossible to get them ready before November; hence we are compelled to issue the present number without the engravings. They shall accompany the January number; those of our subscribers who are in the habit of having the Journal bound at the end of each year, are requested to wait until they receive the first number of the next volume before giving them to the binder.

